# Hepatitis vaccination adherence and completion rates and factors associated with low compliance: A claims-based analysis of U.S. adults

**DOI:** 10.1371/journal.pone.0264062

**Published:** 2022-02-17

**Authors:** Joyce LaMori, Xue Feng, Christopher D. Pericone, Marco Mesa-Frias, Obiageli Sogbetun, Andrzej Kulczycki

**Affiliations:** 1 Janssen Scientific Affairs, Titusville, New Jersey, United States of America; 2 Janssen Medical Affairs, Titusville, New Jersey, United States of America; 3 Department of Health Organization & Policy, University of Alabama at Birmingham, Birmingham, Alabama, United States of America; Centers for Disease Control and Prevention, UNITED STATES

## Abstract

Poor compliance with multi-dose vaccine schedules by adults for whom hepatitis (Hep) A and B vaccines are recommended contributes to major Hep A and B disease burdens among high-risk U.S. adults. Evidence on hepatitis vaccine series adherence, completion, timeliness of completion, and factors associated with these outcomes, is limited and not readily generalizable for U.S. adults. This retrospective, observational study examined adherence, completion, its timeliness, and the impact of sociodemographic and clinical factors on these outcomes among a large, geographically representative sample of U.S. adults. We analyzed the Optum Clinformatics SES administrative claims database (1/1/2010-6/30/2020) for recipients of 2-dose (HepA, HepB2) or 3-dose (HepB3, HepAB) hepatitis vaccines. Adherence was defined as receipt of booster doses within specified assessment periods, per label-recommended schedules. Completion (receipt of all doses) was assessed at 6, 12, 18, and 24 months.The study included 356,828 adults ≥19 years old who were continuously enrolled in a medical benefit plan for one (HepB2), six (HepB3; HepAB), or 18 months (HepA) prior to and following the index date (first observed vaccine dose). Adherence and 24-month completion rates were: HepA (27.0%, 28.4%), HepB2 (32.2%, 44.8%), HepB3 (14.3%, 37.3%), HepAB, (15.3%, 33.8%). Kaplan-Meier completion curves plateaued after about 6 months for HepB2 and about 12 months for HepA, HepB3, and HepAB vaccines. Logistic regression analyses showed risk for low adherence/completion was generally associated with male gender, younger age, Black or Hispanic race/ethnicity, lower educational or household income attainment, and more comorbidities. Adherence and completion rates for all hepatitis vaccine series are low, especially for males, younger adults, those with lower socio-economic status and more comorbidities. To our knowledge, this is the largest claims-based analysis of adherence and completion rates for U.S. adults initiating all currently available HepA and HepB vaccines. Findings may inform hepatitis vaccination programming.

## 1. Introduction

The disease burden of viral hepatitis remains substantial globally and persistent in high income countries, including the USA [1]. The World Health Organization (WHO) estimates that chronic viral hepatitis caused 1.34 million deaths in 2015 from liver cancer, cirrhosis and other conditions [2]. About 325 million people worldwide live with Hepatitis (Hep) B and/or C, the virus types which cause most deaths. Effective vaccines are available to protect against HepA and HepB, whereas HepC is curable by medication, but in most countries, testing and treatment remain beyond reach [3]. In the US, the most common viral types are HepA, B and C. Prevention of these diseases remains challenging, including for the vaccine-preventable liver diseases caused by HepA and B and especially among people with specific risk factors.

The US Centers for Disease Control and Prevention (CDC) has recommended routine pediatric HepA vaccination since 2006 (and for infants in high-risk communities since 1996) and routine HepB vaccination since 1991. HepA and HepB vaccines are currently recommended for adults with risk factors including high-risk sexual practices, intravenous drug use, incarceration, dialysis, insulin injections, and certain chronic medical conditions (e.g., renal or chronic liver disease, hepatitis C or HIV infection), as well as for health care providers and travelers to countries with high prevalence of hepatitis B virus [4]. However, the CDC estimates that in 2019, there were an estimated 37,700, 20,700 and 57,500 acute infections of HepA, B and C, respectively [5]. There has been a particularly large increase in HepA outbreaks, primarily among persons who inject drugs or experience homelessness [6] [7]. Also, although HepB infections have decreased among those <30 years of age due to childhood vaccinations, the incidence of HepB among older adults has increased in recent years [5]. In late 2021, the CDC Advisory Committee on Immunization Practices (ACIP) unanimously voted to recommend universal hepatitis B immunization in all unvaccinated adults aged 59 or younger [8].

Current FDA-approved vaccines for hepatitis include: two HepA vaccines (both are 2-dose), three HepB vaccines (two 3-dose vaccines and a newer, 2-dose vaccine), and a HepA/HepB combination vaccine (3-dose) [4]. Unfortunately, hepatitis vaccination rates for adults have remained consistently low in the US, despite efforts by multiple healthcare organizations to reduce new hepatitis infections and increase vaccination coverage in high-risk adults [9]. As all hepatitis vaccines are multi-dose, their effectiveness is also hampered by poor compliance with recommended dosing schedules, particularly for underserved and hard-to-reach populations. National Health Interview Survey (NHIS) data from 2018 showed that only 11.9% and 30.0% of adults aged ≥19 years had completed a HepA or HepB vaccine series, respectively [10]. Real world studies using administrative claims data have reported somewhat higher completion rates for adults ≥19 years, approximately 32% for HepA and 31–40% for HepB [11, 12]. However, data on adherence to and completion of the vaccine series, and timeliness of series completion, are all limited, despite their importance to optimizing disease protection and for broader health programming.

Additionally, factors associated with adhering to and completing the multi-dose HepA and Help B are inadequately monitored and understood. Importantly, there is growing evidence for significant racial/ethnic and socio-economic disparities in hepatitis vaccination rates among U.S. adults [13–15]. However, detailed information on such disparities is lacking because many studies use survey data, which are subject to recall and nonresponse bias [13–15]. Most recent studies have utilized administrative claims or electronic health records, but these sources often lack detailed sociodemographic data. In addition, there is a lack of data on completion rates for the new 2-dose HepB vaccine (approved in November 2017) and few studies examine the impact of socioeconomic factors or other patient, provider, or payer variables [16]. Lastly, studies of multi-dose vaccines typically focus on initiation of vaccination course and completion (proportion of recipients receiving all doses) rather than adherence (proportion of recipients receiving subsequent doses within a certain period of time), which is also an important parameter to consider in the context of controlling disease [17].

These factors underscore the need for more recent evidence on multi-dose vaccines, including the influence of patient characteristics on adherence and completion, to better inform initiatives to improve vaccination rates and outcomes, particularly for high risk and underserved populations. Therefore, the aims of this study were to assess rates of adherence and completion, and time to completion, among adults who initiated vaccines for hepatitis. Further, we evaluated the impact of sociodemographic and clinical factors on the rates of adherence and completion to multi-dose hepatitis vaccine schedules.

## 2. Materials and methods

### 2.1 Data source

This study used the Optum Clinformatics Data Mart–Socio-economic Status (SES) database, an adjudicated administrative health claims database for members with private health insurance. The population is primarily representative of U.S. commercial claims patients (0–65 years old) with some Medicare patients (65+ years old); however, ages are capped at 90 years [18, 19]. It includes data captured from administrative claims processed from inpatient and outpatient medical services and prescriptions as dispensed, as well as results for outpatient lab tests processed by large national lab vendors who participate in data exchange with Optum. Optum Clinformatics Data Mart SES provides socio-economic information for members with both medical and pharmacy coverage and location information for patients at the U.S. Census Division level.

### 2.2 Study design

This retrospective, observational study assessed claims data from January 1, 2010 to June 30, 2020 to examine adherence to and completion of the recommended vaccine-specific schedules among adults vaccinated with HepA (2 doses), HepB2 (2 doses), HepB3 (3 doses) or HepAB (3 doses). The index date was the first observed dose of each vaccine during the study period. The baseline period was defined as one month prior to the index date (HepB2); six months prior (HepB3; HepAB); and 18 months prior to the index date (HepA). The follow-up period was defined as one month after the index date (HepB2); six months after (HepB3/HepAB); and 18 months after the index date (HepA). Current Procedural Terminology (CPT) codes were used to identify prescriptions for each vaccine (S1 Table).

### 2.3 Study population

The study population was composed of adults aged ≥19 years at the index date who had a prescription for the HepA (Vaqta, Havrix), HepAB (Twinrix), HepB2 (Heplisav B), or HepB3 (Recombivax HB, Engerix B) vaccine and were continuously enrolled in a medical benefit plan during the baseline and follow-up periods for each study vaccine (Table 1). For the analyses of completion rates, an additional inclusion criterion of continuous enrollment for 24 months post-index was applied, in order to capture completion events that occurred after the label-recommended period.

**Table 1:** Recommended adult dose schedules for HepA, HepB2, HepB3 and HepAB.

Vaccine Name	Type	Age	Doses	Dose 2 timing	Dose 3 timing	Assessment Period
Vaqta	HepA	19+ yrs	2	6–18 mo	-	6–18 mo
Havrix	HepA	19+ yrs	2	6–12 mo	-	6–18 mo
Twinrix	HepAB	19+ yrs	3	1 mo	6 mo	At 1 mo and 6 mo
Recombivax HB	HepB	19+ yrs	3	1 mo	6 mo	At 1 mo and 6 mo
Heplisav B	HepB	19+ yrs	2	1 mo	-	At 1 mo
Engerix B	HepB	19+ yrs	3	1 mo	6 mo	At 1 mo and 6 mo

Hep = hepatitis; mo = months; yrs = years.

Adults who received the relevant vaccine during baseline (i.e., pre-index period), received the accelerated HepAB dosing schedule (i.e., >3 doses) or initiated a second or third dose of vaccine prior to the label-recommended dosing visit were excluded. Patients with immunosuppression or undergoing dialysis (CPT codes: 90747, 90740) were also excluded, since it is generally recommended that these patients receive additional hepatitis B doses [12, 20].

### 2.4 Variables

Variables included index year; sociodemographic characteristics (gender, age at index date, race, Hispanic ethnicity, major geographic region, household income range, education level, and insurance type); and clinical characteristics (provider specialty type, Quan-Charlson Comorbidity Index [CCI; see S2 Table], chronic comorbidities [diabetes, liver disease, renal diseases], presence of inpatient visits or emergency room [ER] visits at baseline).

### 2.5 Outcomes

Rate of adherence, rate of completion (for distinct follow-up times described below), and time-to-completion were determined for HepA, HepB2, HepB3 and HepAB. Relationships between sociodemographic/clinical factors and the outcomes of adherence or completion were also evaluated among those who had initiated these vaccines. Lastly, variations in the rates of adherence and completion of these vaccines were assessed across specific geographic regions in the U.S.

### 2.6 Adherence to vaccine schedules

Adherence (yes/no) was defined as the receipt of the second or third dose (if applicable) within the specified assessment periods, which were based on each vaccine’s label-recommended dose schedule, as recommended by the Advisory Committee on Immunization Practice (Table 1) [21]. If the minimum time between the two doses was violated, the latter dose was considered invalid [22]. The adherence rate for each vaccine type and dose was calculated as follows:

$$Adherence\;rate=\frac{Number\;of\;enrollees\;adhering\;to\;the\;recommendations}{Number\;of\;enrollees\;with\;prescription\;for\;vaccine\;first\;dose}x\;100\%$$

Adherence was defined according to the following criteria: 1) HepA vaccine—individuals who received two doses of HepA vaccines with the second dose administered between 6 months (183 days) and 18 months (549 days) following the index date; 2) HepB2—individuals who received two doses of HepB vaccines with the second dose administered at one month (31 days) following the index date (with a 4-day grace period prior to the minimum dose spacing window allowed); 3) HepB3—individuals who received three doses of HepB vaccine with the second dose administered at one month (31 days) following the index date and third dose administered at 6 months (183 days) following the index date (4-day grace period prior to the minimum dose spacing window allowed); and 4) HepAB—individuals who received three doses of HepAB vaccine with the second dose administered at one month (31 days) following the index date and third dose administered at 6 months (183 days) following the index date (4-day grace period prior to the minimum dose spacing window allowed).

### 2.7 Completion of vaccine schedules

Continuous enrollment of medical plans for 24 months was essential across all groups to understand the completion rates across different time points. The measure used to assess the completion rate for each vaccine and dose within the time frame of 6, 12, 18, and 24 months after the first dose (i.e., index date) was defined as follows:

$$Completion\;rate=\frac{Number\;of\;enrollees\;completing\;vaccine\;series\;within\;specified\;time}{Number\;of\;enrollees\;with\;prescription\;for\;vaccine\;first\;dose}x\;100\%$$

### 2.8 Data analysis

Patient characteristics and clinical/behavioral characteristics were assessed at the index date and/or baseline. Frequencies and percentages for categorical variables and means and standard deviations (SD) for continuous variables were summarized. A p-value of <0.05 was considered statistically significant. All data analyses were conducted using SAS Enterprise Guide 7 (SAS Institute, Cary, NC).

#### 2.8.1 Adherence to/completion of vaccine schedules

Each vaccine was analyzed separately. Frequencies and percentages (adherence and completion rates) were presented for all vaccines, classified by vaccine type and number of label-recommended doses. Cumulative proportions of enrollees completing each vaccine type were plotted by time. Kaplan-Meier (KM) survival methods were used to assess time to completion of the final dose and the proportion of individuals who completed the final dose. Using the KM method allowed for right-censoring of records for enrollees with no documented subsequent doses in the claims, which enabled inclusion of the full sample in the analyses (i.e., 24 months of continuous enrollment was not required). In these analyses, individuals were censored at the end of continuous enrollment or the end of the data assessment period.

#### 2.8.2 Impact of sociodemographic/clinical factors on vaccine adherence and completion

The adherent (or completion) group was compared with the non-adherent (or non-completion) group using Chi-square tests. All statistical tests were two-sided, and significance was considered at p<0.05 for all tests. Logistic regressions were used to assess the impact of the sociodemographic and clinical/behavioral related variables on adherence to recommended vaccine schedules for each vaccine and dose. The impact on completion was assessed using a 24-month assessment period.

#### 2.8.3 Sensitivity analyses

For adherence, we performed a sensitivity analysis by extending the baseline period to 12 months for the HepB2, HepB3, and HepAB cohorts (consistent with the HepA cohort). Thus, these analyses included only individuals with 12 months continuous eligibility prior to first vaccine dose.

### 2.9 Research ethics

All study data were accessed with protocols compliant with U.S. patient confidentiality requirements, including the Health Insurance Portability and Accountability Act of 1996 (HIPAA). The study was exempt from all 45 CFR part 46 requirements because it used existing fully de-identified data and the patients and investigators could not be identified, directly or through identifiers linked to subjects.

## 3. Results

### 3.1. Patient attrition and overall sample size

**Fig 1:**
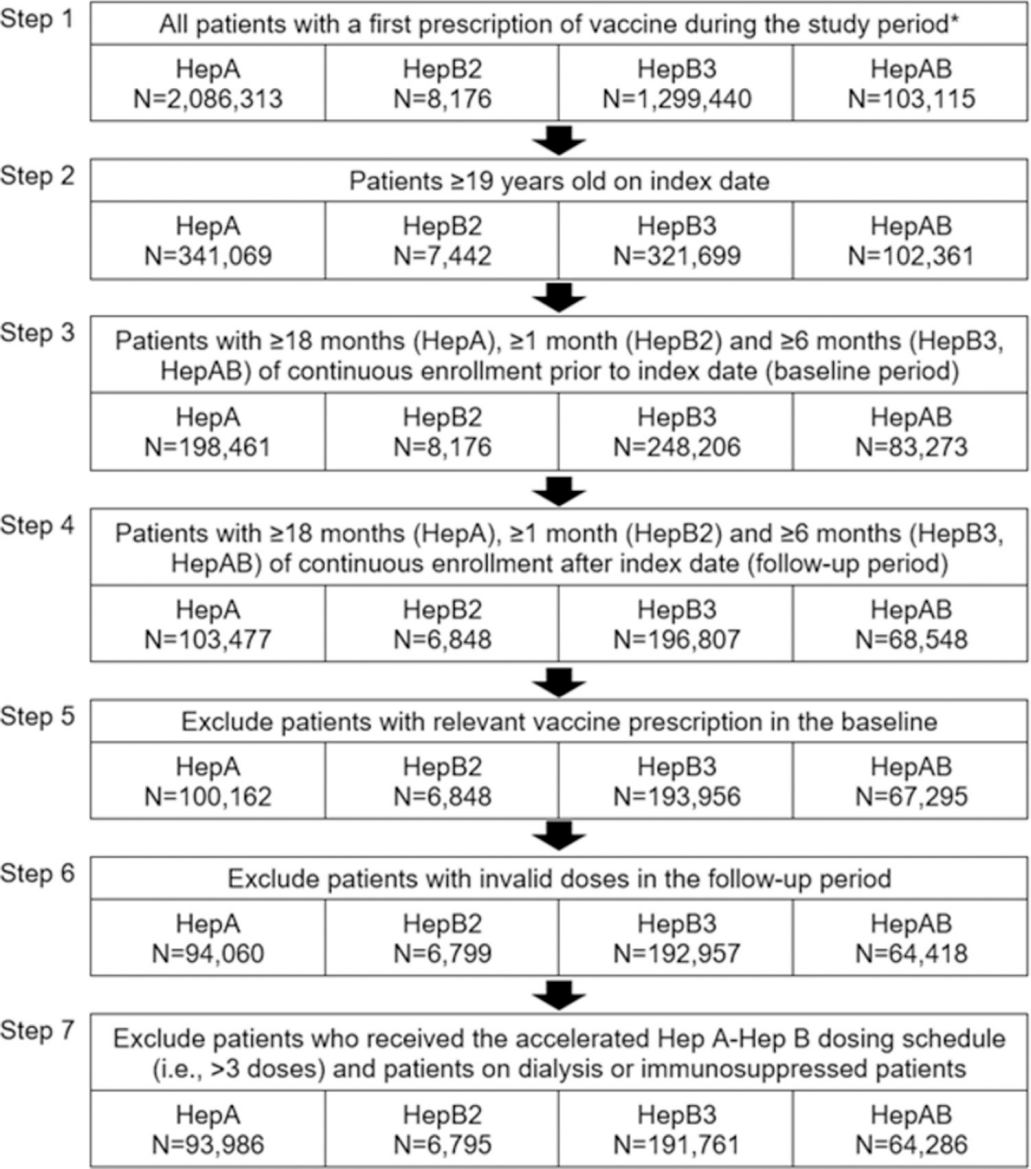
Patient identification flowchart. *Study period for HepA: 1/01/2011 to 6/30/2020; HepB2: 11/7/2017 to 6/30/2020; HepB3 and HepAB: 1/01/2011 to 06/30/2020. **Note:** HepB2 became available in the U.S. on 11/7/2017.

Fig 1 shows the derivation of the final study samples, per the inclusion/exclusion criteria. After applying all inclusion/exclusion criteria, the number of patients included in the study who initiated each vaccine were: HepA, 93,986; HepB2, 6,795; HepB3, 191,761; HepAB, 64,286. The number of patients with sufficient follow-up time to assess 24-month completion rates were: HepA, 75,561; HepB2, 134; HepB3, 99,560; HepAB, 34,925 (S3 Table). The majority of attrition was due to the requirements for adequate baseline and follow-up time; whereas exclusion of patients who received the accelerated HepAB dosing schedule, were undergoing dialysis or were immunosuppressed (see Steps 5–7 in Fig 1) reduced the study sample by approximately 1%.

Demographic and clinical characteristics of all eligible vaccine recipients (i.e., the cohorts used to assess adherence) are presented in Table 2. Gender distribution was approximately equal in each cohort (48–54% female). Mean age ranged from 44 to 51 years. Cohorts ranged in race/ethnic composition from 52–73% white, 7–13% Hispanic, 6–10% black, 6–10% Asian, and 5–21% with unknown/other race, which is broadly consistent with the overall US population [23]. The HepA cohort had the highest proportion with white race (73.2%) and the lowest proportion with unknown/other race (5.1%). The HepB2 cohort had the lowest proportion with white race (51.7%) and the highest proportion with unknown/other race (21.3%). Across the various vaccine cohorts, the majority of initiators lived in a neighborhood with median household income ≥$40,000 (55–78%), and education beyond a high school diploma (64–86%). The HepA cohort had the highest proportion with median household income ≥$100,000 (49.1%) and education beyond a high school diploma (85.7%). The HepB2 cohort had the lowest proportion with median household income ≥$100,000 (28.5%) and education beyond a high school diploma (64.4%). The greatest proportion of the study population lived in the South and the smallest proportion was from the Northeast, consistent with the geographic distribution of the Optum database [18, 19].

**Table 2:** Socio-demographic and clinical characteristics of vaccine recipients (by vaccine series type).

	HepA (2-dose)		HepB (2-dose)			HepB (3-dose)		HepAB (3-dose)	
	N	%	N	%		N	%	N	%
Overall	93986		6795			191761		64286	
Gender									
Male	43619	46.4	3551	52.3		88423	46.1	29546	46.0
Female	50367	53.6	3244	47.7		103338	53.9	34740	54.0
Age group									
18–39	39193	41.7	1753	25.8		71329	37.2	18748	29.2
40–64	41533	44.2	3152	46.4		88938	46.4	38302	59.6
65–74	9750	10.4	1326	19.5		24252	12.6	5993	9.3
≥75	3508	3.7	564	8.3		7242	3.8	1243	1.9
Race/ethnicity									
Asian	7435	7.9	465	6.8		18389	9.6	3610	5.6
Black	6002	6.4	635	9.3		18394	9.6	6521	10.1
Hispanic	6770	7.2	751	11.1		24089	12.6	6074	9.4
White	68993	73.4	3497	51.5		115702	60.3	43980	68.4
Unknown/other	4786	5.1	1447	21.3		15187	7.9	4101	6.4
Region									
Northeast	11341	12.1	574	8.4		25725	13.4	5217	8.1
Midwest	30270	32.2	1301	19.1		46914	24.5	14423	22.4
South	26994	28.7	2967	43.7		69793	36.4	29708	46.2
West	25224	26.8	1945	28.6		48802	25.4	14870	23.1
Unknown	157	0.2	8	0.1		527	0.3	68	0.1
Household income									
<$40k	8383	8.9	995	14.6		28508	14.9	8478	13.2
$40k-60k	7410	7.9	628	9.2		20199	10.5	6588	10.2
$60k-100k	18695	19.9	1193	17.6		38901	20.3	13731	21.4
$100k +	46103	49.1	1936	28.5		66992	34.9	24855	38.7
Unknown	13395	14.3	2043	30.1		37161	19.4	10634	16.5
Education level*									
≤ High School	11074	11.8	1139	16.8		36715	19.1	12320	19.2
> High School	80528	85.7	4377	64.4		144303	75.3	49184	76.5
Unknown	2384	2.5	1279	18.8		10743	5.6	2782	4.3
Insurance type									
Commercial	81915	87.2	4613	67.9		155142	80.9	57253	89.1
Medicare	12071	12.8	2182	32.1		36619	19.1	7033	10.9
CCI condition**									
0	72412	77.0	5279	77.7		141767	73.9	49968	77.7
1–2	16034	17.1	986	14.5		33438	17.4	9634	15.0
≥3	5540	5.9	530	7.8		16556	8.6	4684	7.3
ER visit—baseline	23261	24.7	300	4.4		49994	26.1	9010	14.0
In patient -baseline	8391	8.9	171	2.5		26857	14.0	3775	5.9
Provider type for first dose									
Family practice	42985	45.7	2748	40.4		90235	47.1	27452	42.7
Internal medicine	20859	22.2	1664	24.5		44578	23.2	12932	20.1
Nursing	3014	3.2	275	4.0		5372	2.8	1789	2.8
Pharmacy	453	0.5	282	4.2		7997	4.2	2724	4.2
Infectious disease	3477	3.7	206	3.0		3077	1.6	2156	3.4
Others	23194	24.7	1620	23.8		40501	21.1	17233	26.8
Unknown	4	0.0	0	0.0		1	0.0	0	0.0

^α^census block level.

^β^Number of conditions from the Quan-Charlson Comorbidity Index (S2 Table).

CCI = Charlson Comorbidity Index; ER = emergency room; Hep = hepatitis.

The majority of adults who initiated a vaccine series had no Quan-Charlson Comorbidity Index (CCI) conditions (74–78% across cohorts), with 15–17% having 1–2 CCI conditions and 6–9% having ≥3 CCI conditions. There was also little variation across cohorts in terms of healthcare providers administering the vaccine, with most initial vaccine doses being administered by either a family practice (40–47%) or internal medicine (20–25%) physician. Nurses, pharmacists, and infectious disease physicians each administered approximately 3–4% of initial vaccine doses for each cohort, except that pharmacists performed very few initial doses for HepA (<1%). All other healthcare providers administered from 21–27% of initial doses across the vaccine series. Few adults had a baseline ER-visit (14–26%) or in-patient visit (6–14%) among the HepA, HepB3, or HepAB cohorts (18-, 6-, and 6-month baseline period respectively), with these proportions being even lower (4.4% and 2.5%, respectively) for the HepB2 cohort (1-month baseline period).

The cohorts used to assess completion for each vaccine were smaller (due to 24-month required follow-up time), but their overall distribution of demographic and clinical characteristics was very similar to those of the adherence cohorts (S4 Table).

### 3.2. Vaccine completion rates

Completion rates using assessment periods of various lengths are presented in Table 3. For HepA, the overall completion rate was 26.6% at 18 months and 28.4% at 24 months. For HepB2, the overall completion rate was 37.3% at 6 months and 44.8% at 24 months. Among 3 dose vaccines, the overall completion rate at 6 months was 11.1% and 7.4% for HepB3 and HepAB, respectively, and at 24 months it was 37.3% and 33.8%, respectively.

**Table 3:** Proportion of individuals who completed the vaccine series at 6, 12, 18 and 24 months after first dose.

	Assessment Period							
	6 months		12 months		18 months		24 months	
	N	%	N	%	N	%	N	%
**HepA (2-dose) N = 75561**	N/A	N/A	N/A	N/A	20075	26.6	21445	28.4
**HepB (2-dose) N = 134**	50	37.3	57	42.5	58	43.3	60	44.8
**HepB (3-dose) N = 99560**	11051	11.1	33054	33.2	35941	36.1	37180	37.3
**HepAB (3-dose) N = 34925**	2584	7.4	10582	30.3	11455	32.8	11820	33.8

Hep = hepatitis; N/A = not applicable.

### 3.3. Time to completion

**Fig 2:**
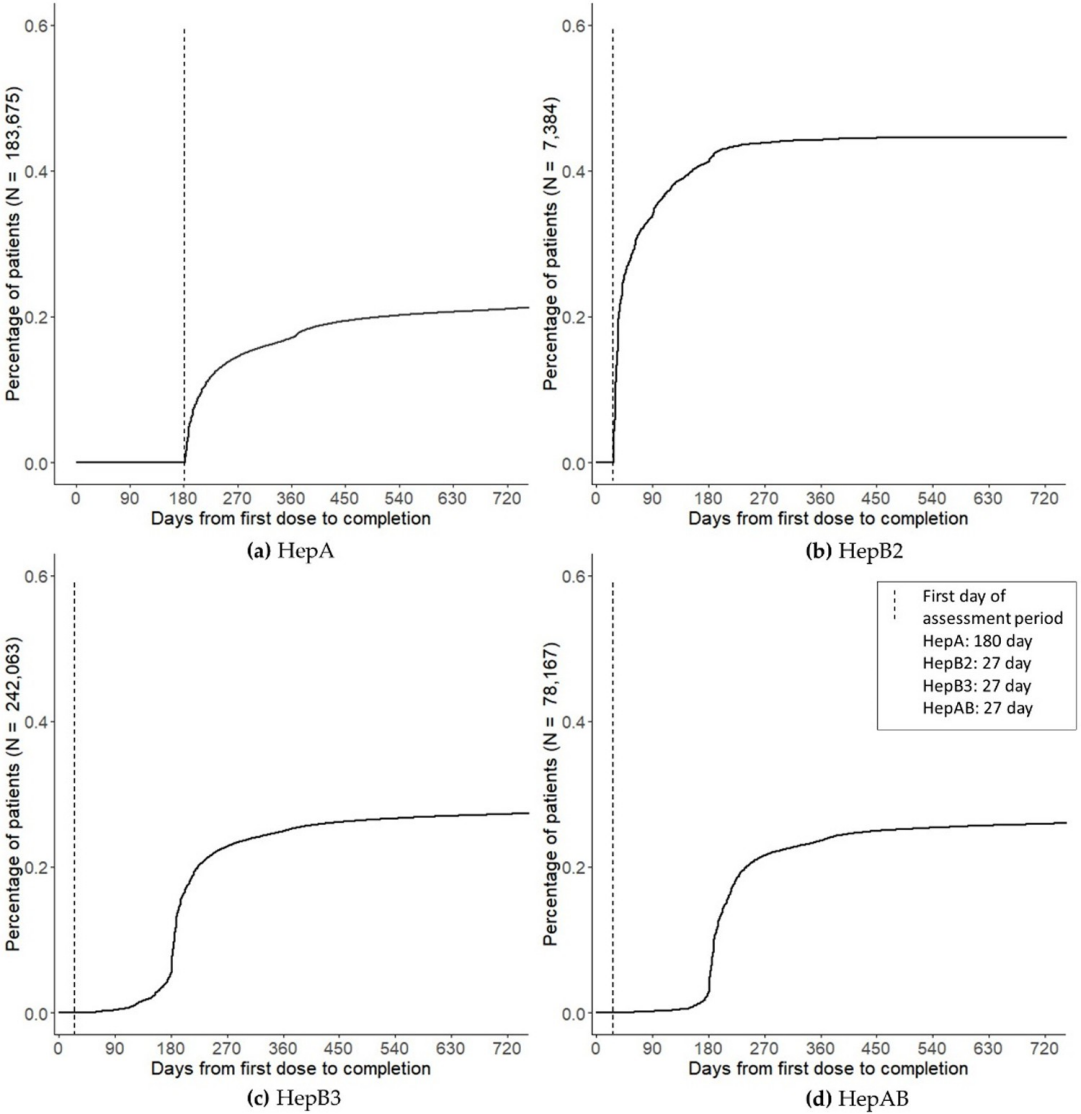
Cumulative proportion of individuals who completed the vaccine series, by time (in days) since first dose, for: (a) HepA (b) HepB2 (c) HepB3 (d) HepAB.

Kaplan-Meier curves were generated to show cumulative completion over time for each vaccine. Among 2 dose vaccines, the HepA completion curve did not change substantially after about 12 months, whereas the curves for HepB2 did not change substantially after about 6 months. Among 3 dose vaccines (HepB3, HepAB), the completion curves did not change substantially after about 12 months (Fig 2).

### 3.4. Vaccine adherence rates among all vaccine initiators

Overall, adherence (receipt of the second and third dose [if applicable] within the specified assessment periods) was approximately twice as high for the 2 dose vaccines (HepA, 27.0%; HepB2, 32.2%) compared to the 3 dose vaccines (HepB3, 14.3%; HepAB, 15.3%) (Table 4).

**Table 4:** Adherence rates among US adults who initiate vaccination for Hepatitis A or B.

	Hep A (2-doses)	Hep B (2-doses)	Hep B (3-doses)	Hep AB (3 doses)
	N = 93,986	N = 6,795	N = 19,1761	N = 64,286
Overall, (%)	27.0	32.2	14.3	15.3
Gender				
Male, (%)	26.3	31.3	13.9	13.4
Female, (%)	27.5	33.1	14.7	17.0
Age group				
18–39	22.0	32.3	11.6	13.1
40–64, (%)	32.2	32.0	15.7	17.8
65–74, (%)	26.4	31.2	16.6	8.9
≥75, (%)	22.3	34.8	17.0	6.1
Race/ethnicity				
Asian, (%)	27.4	33.8	18.4	14.2
Black, (%)	23.3	35.7	11.6	13.5
Hispanic, (%)	24.2	24.4	12.0	12.9
White, (%)	27.5	33.7	14.6	16.3
Unknown/other, (%)	26.7	30.3	13.5	12.5
Region				
Northeast, (%)	25.4	40.4	16.6	13.5
Midwest, (%)	28.6	32.7	14.1	14.2
South, (%)	26.7	31.6	13.9	18.2
West, (%)	26.1	30.1	13.9	11.5
Household income				
<$40k, (%)	27.1	28.6	12.8	13.2
$40k-60k, (%)	26.9	35.9	14.2	15.3
$60k-100k, (%)	28.1	36.9	15.4	17.2
$100k +, (%)	26.9	33.2	15.4	16.1
Unknown, (%)	25.3	31.1	12.4	12.7
Education level α				
≤ High School, (%)	30.0	29.5	13.1	16.3
> High School, (%)	26.5	33.4	14.7	15.4
Unknown, (%)	26.5	30.2	13.0	10.9
Insurance type				
Commercial, (%)	27.4	32.1	14.0	16.7
Medicare, (%)	24.1	32.3	15.7	4.5
CCI condition β				
0, (%)	26.1	31.9	13.9	15.5
1–2, (%)	29.7	32.2	15.6	15.9
≥3, (%)	30.2	34.5	15.0	12.3
ER visit–baseline	27.3	30.3	15.4	15.4
In patient -baseline	27.0	29.8	13.0	14.3
Provider type for first dose				
Family practice, (%)	28.0	29.3	13.4	12.7
Internal medicine, (%)	28.5	33.7	18.2	15.5
Nursing, (%)	23.7	33.1	12.2	14.6
Pharmacy, (%)	34.9	33.7	15.0	17.0
Infectious diseases, (%)	29.0	41.7	13.8	18.7
Others, (%)	23.6	33.8	12.1	18.8

^α^census block level.

^β^ Number of conditions from the Quan-Charlson Comorbidity Index (S2 Table).

Note

*** p<0.001

** p<0.01

*p<0.05 from Chi square test.

Similar results were observed in the sensitivity analyses which required all individuals to have 12 months continuous eligibility prior to their first vaccine dose: HepB2 (32.8%), HepB3 (14.8%), HepAB (15.9%) (S5 Table).

### 3.5. Demographic and socioeconomic correlates of vaccine adherence rates

Adherence rates for each vaccine, by demographic and socioeconomic factors, are presented in Table 4. For all four vaccine cohorts, adherence rates were consistently higher among females and those living in a neighborhood with median household income of $60–100,000 (and as high for the HepB3 cohort for those with a median household income ≥$100,000). Adherence rates were generally lowest for adults with low, high, or unknown median household income levels, living in the West, and with no CCI conditions.

For HepA, adults 18–39 years old had the lowest rate of adherence (22.0%) among all age groups, followed closely by those aged ≥75 years (22.3%). Among racial/ethnic subgroups, adherence rates were lowest among individuals who were Black (23.3%) or Hispanic (24.2%). Adherence was also lower for men (26.3%), for adults with Medicare insurance (24.1%), and for those living in the Northeast (25.4%). Additionally, adherence rates were lowest for adults with a median household income of $40–60,000 or ≥$100,000 (26.9% each), and with education beyond a high school diploma (26.5%), as well as those with unknown household income or educational status (Table 4). Adherence was also lowest for individuals with no CCI conditions (26.1%), those who had an ER visit (27.3%) or were in-patients at baseline (27.0%), and those who received their first vaccine dose from a nursing provider (23.7%).

For HepB2, adherence rates were lowest for men (31.3%), adults 65–74 years old (31.0%), Hispanics (24.4%), adults with a high school diploma or less (29.5%), and those with commercial insurance (32.1%). Adherence rates were also lowest among those living in the West (30.1%), in a neighborhood with a median household income <$40,000 (28.6%), and those who received their first vaccine dose from a family practitioner (29.3%).

For HepB3, adherence rates were lowest among the youngest adults, aged 18–39 years (11.6%); adults with Black (11.6%) or Hispanic race/ethnicity (12.0%); adults living in a neighborhood with a median household income <$40,000 (12.8%), in the South or West regions (13.9% each); those with commercial insurance (14.0%); and adults with a high school diploma or less (13.1%).

For HepAB, adults ≥75 years old (6.1%), Hispanic (12.9%) and Black adults (13.5%), and those with unknown or other race/ethnicity had the lowest rates of adherence. Regarding other characteristics, adherence rates were also lowest for those living in a neighborhood with a median household income <$40,000 (13.2%) and those with more than a high school diploma (15.4%) or with unknown educational status (10.9%). Adults with commercial insurance (16.7%) were over 3 times more likely to adhere to the recommended vaccine schedule than were those with Medicare (4.5%).

### 3.6. Demographic and socioeconomic correlates of completion

Completion rates (at 24 months) for each vaccine, by demographic and socioeconomic factors are presented in S3 Table. Differences in completion rates between demographic and socioeconomic groups generally followed similar trends as for adherence, with some exceptions.

Among HepA initiators, the differences only related to educational attainment, with completion rates lowest among those with a bachelor’s degree or higher (32.5%). There was more variation among HepB2 initiators, but the sample size was much lower (n = 134) and does not allow for meaningful comparisons between correlates of HepB2 completion vs adherence. Among HepB3 and HepAB initiators, the demographic and socioeconomic correlates of completion were the same as for adherence rates for all variables, except for those who received their first vaccine dose at a nursing facility (31–32%) (S3 Table).

### 3.7. Adherence and completion rates for initiators with chronic comorbidities

Adherence and completion rates (at 24 months) for HepA, HepB3 and HepAB initiators with chronic comorbidities (diabetes, liver disease, renal disease) are presented in S6 Table (the HepB2 sample size was too low to include in this analysis). For the HepA and HepB3 cohorts, adherence and completion rates were generally unchanged or higher among those with chronic comorbidities whereas for the HepAB cohort, adherence and completion rates were generally unchanged or lower.

### 3.8 Logistic regression analyses of adherence and completion

**Fig 3:**
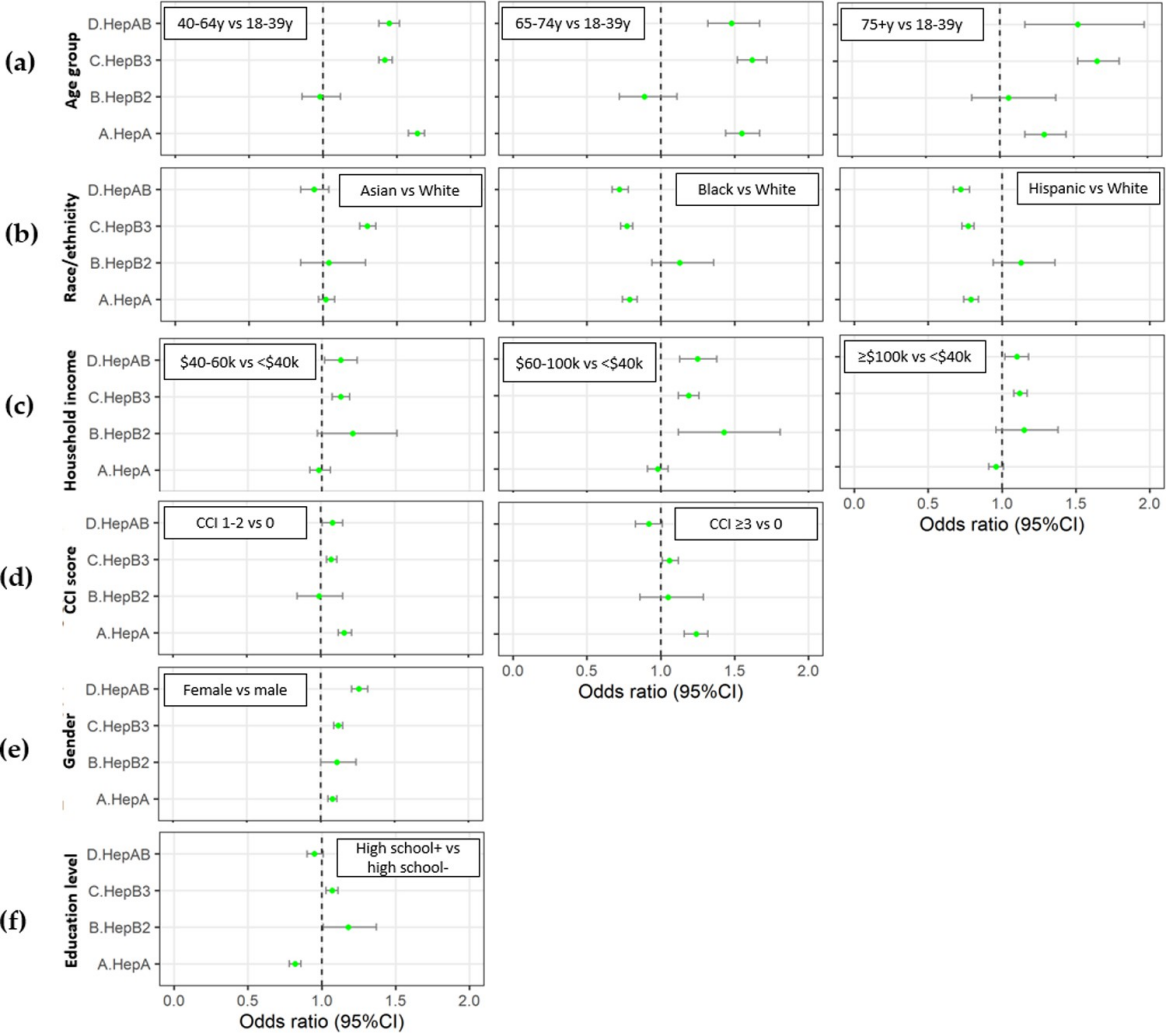
Impact of sociodemographic and clinical/behavioral variables on adherence to Hepatitis A and B vaccine schedules (a) Age (b) Race (c) Income (d) CCI score (e) Gender (f) Education. CCI: Charlson Comorbidity Index; CI: Confidence Interval. Right side of the dashed line indicates greater odds of adherence vs the reference subgroup.

Odds ratios from a regression analysis of factors associated with adherence to each vaccine are presented in Fig 3A–3F. Female gender was consistently associated with better adherence across all four vaccine cohorts. For all cohorts except HepB2, adherence rates were significantly lower among adults who were younger (aged 18–39 years), Black, Hispanic, or insured by Medicare, as compared with adults who were older, white, or had Commercial insurance. Higher education level was associated with better adherence for HepB2 and HepB3, but the opposite was true for HepA. In addition, higher median household income was associated with better adherence for all vaccines except for HepA. Lastly, the presence of 1 or more comorbidities at baseline was associated with better adherence for HepA and HepB3, whereas adherence for HepAB was greater for adults with 1–2 comorbidities as compared with adults with 0 or ≥3 comorbidities. Full results from the logistic regression analysis of adherence are presented in S7 Table.

Results from the logistic regression analysis of completion rates for each vaccine (at 24 months) are presented in S8 Table. Female gender, age >39 years, Commercial insurance, higher median household income, and higher number of comorbidities were generally associated with greater completion rates, with the exceptions noted above for adherence. In contrast to the results for adherence, white race was associated with lower HepA completion rates as compared with Black race or Hispanic ethnicity, and higher education level was associated with lower HepAB completion rates. Results from the logistic regression analyses of completion for HepB2 should be interpreted with caution, due to low sample size of this cohort.

## 4. Discussion

Safe and effective vaccines have significantly reduced the reported incidence of HepA and HepB cases, but infections persist in high-risk and other adults. There is limited information on HepA and HepB vaccine series adherence and completion among adults, and on factors that influence compliance with the required multi-dose vaccine schedules, which present greater challenges than do single-dose immunizations. Among a large, geographically representative sample of all U.S. commercial claims adult patients aged ≥19 and including some Medicare patients, we observed low adherence (14–32%) and completion rates (28–45% at 24 months) for all multi-dose hepatitis vaccines. Completion rates were higher when adults without 24-months of eligibility were retained in the analysis, but these rates generally plateaued after about 6 months for HepB2 and about 12 months for HepA, HepB3, and HepAB vaccines, consistent with previous reports in the literature [11, 12]. Taken together, these results indicate that a large proportion of patients who complete their vaccination series do not do so within the recommended time frame. Furthermore, adherence and completion rates varied by patient demographic, socioeconomic and clinical characteristics. Overall, younger age, Black/Hispanic race/ethnicity, number of comorbidities, and lower household income or educational status tended to be associated with lower vaccine series adherence and completion rates. However, for the HepA and HepB3 cohorts, adherence and completion rates tended to be higher among those with high-risk comorbidities (diabetes, liver disease, and renal disease).

To our knowledge, this study is the first to use the Optum Clinformatics database to examine adherence and completion rates of the various hepatitis A and B vaccines. It is also the first to use a geographically diverse administrative claims database to examine socioeconomic factors associated with both adherence and completion. In contrast, existing studies have tended to be restricted to higher risk populations [14, 15, 24, 25] or localized geographic areas [16]. A recent study using self-reported NHIS survey data found poor coverage rates for hepatitis vaccines and although that study assessed sociodemographic correlates, information on adherence was not provided [13]. Furthermore, while some studies have used administrative claims databases to assess completion and, less frequently, adherence rates of hepatitis vaccines among adults [11, 12], the current study is novel due to its analysis of adherence and completion of all hepatitis vaccines, including the recently introduced 2-dose hepatitis B vaccine, as well as their associated factors.

Our results confirm and extend the body of evidence from previous real-world administrative claims studies which have shown that low completion rates for hepatitis vaccines are associated with risk factors such as age, race, income level, region, and specific comorbidities [11, 12, 24, 26]. Bruxvoort et al recently reported higher completion rates for 2-dose or 3-dose Hepatitis B vaccines among those who were older, female, white, Asian or Pacific Islander, living in a census block with higher median income or with >75% of individuals with high school education or higher [16]. However, their analysis utilized data from Kaiser Permanente, an integrated healthcare delivery network in Southern California, whereas our study used a large claims database that is more representative of the entire US [18].

Racial/ethnic/economic vaccination disparities in the U.S. have been observed to varying degrees for other multi-dose vaccines, including those for human papillomavirus (HPV), pneumococcus, and zoster [10, 15, 27, 28]. Such disparities have been attributed to lower awareness of disease risk factors, lack of insurance coverage, concerns about cost, and low confidence in vaccines or the healthcare system [24, 27, 29] Lower vaccine completion rates for disadvantaged minorities and people in low-income households are also seen among children and adolescents [30]. This is despite the CDC’s Vaccines for Children (VFC) program, a federally funded program that provides vaccines at no cost to children whose parents/guardians may not otherwise be able to afford them [31], and which has minimized financial barriers to starting the HPV vaccination series among low-income and vulnerable minority adolescents [30, 31]. However, our research has focused on multi-dose vaccines recommended for adults without ready access to such support. In ongoing work, we are studying factors associated with completion and adherence for the recently approved recombinant zoster vaccine, also using a large, geographically representative database.

A recent study reported that challenges associated with completion of the 3-dose Hepatitis B vaccine included limited ability of clinics to conduct reminders for booster doses due to frequent changes in patients’ contact information [24]. Such challenges may be more common in individuals with lower income or education level, consistent with our results showing lower adherence and completion among these groups. However, in contrast to other hepatitis vaccines, we found lower adherence and completion for Hepatitis A among adults with higher household incomes. This may be partly related to a relatively low Hepatitis A vaccine completion rate among international travelers [32], who are much more likely to be higher income adults.

Adherence and completion rates were greater (and time to completion generally shorter) for the HepB2 vaccine than other hepatitis vaccines, likely reflecting its relatively short duration (1 month) between the first and second doses. We also observed that in contrast to the other vaccines, greater adherence to the 2-dose Hepatitis B vaccine was not predicted by older age, Commercial insurance, or white race. Interestingly, we observed a lower mean age, household income, and lower proportion of adults with Commercial insurance or white race in our 2-dose Hepatitis B vaccine cohort, suggesting this vaccine is being prescribed to a somewhat different population of adults. More research on adherence and completion rates for this newly approved 2-dose Hepatitis B vaccine is warranted, especially to support initiatives to increase hepatitis vaccinations, as reflected in the national plan towards elimination of viral hepatitis [33] and in the US Healthy People 2030 goals [9]. Also, since the ACIP recently voted to recommend universal hepatitis B immunization in all unvaccinated adults aged 59 or younger, this study serves as a valuable baseline for future studies as it captures the situation shortly before this recommendation was made [8].

From a public health perspective, optimal protection for at-risk populations using multi-dose vaccines requires not only eventual completion, but also adherence to the recommended schedule (i.e., timely completion) [17]. Additionally, recent studies have shown that vaccines with fewer doses are more cost effective, as their logistical simplicity contributes to better completion, adherence, and thus, better protection [34, 35]. Indeed, despite unprecedented initiatives to encourage vaccination, recent reports show that approximately 10% of people in the US who initiate a 2-dose COVID-19 vaccine are non-adherent (i.e., do not receive a 2^nd^ dose within 42 days) [36], and the majority of those who are non-adherent do not complete the series [37]. Our study provides valuable data through the last decade and into the first 6 months of the ongoing COVID-19 pandemic, which may also inform future research into the impact of pandemics on immunization rates. Although motivation to receive the influenza vaccine may have increased during the COVID-19 pandemic [38], coverage of many other vaccines has been negatively impacted by the pandemic, among both adults and children [39, 40]. Similarly, it is likely that adherence and completion rates for multi-dose hepatitis vaccines have decreased during the COVID-19 pandemic, especially among high-risk individuals who are probably even more susceptible to pandemic-related challenges in seeking healthcare than they are under more conventional circumstances [24]. Pandemic induced hardships are believed to underpin a surge in drug-overdose deaths in the U.S. in 2020 [41], which may foreshadow increases in hepatitis cases.

The current study is subject to limitations. Its observational nature prevents interpretation of between-group differences in adherence or completion as causal effect estimates. Also, the administrative claims data were collected for the purpose of facilitating payment for healthcare services and may contain potential coding errors or omissions, although there are no reasons to expect these to be unduly large for immunizations. In addition, some variables (race, income, education) were not captured directly, but were derived from Optum’s demographic-based analytic models or geographic location. Claims data also do not allow us to determine the reason why an individual did not complete their vaccine series or properly adhere to the schedule. Lastly, this study included only people who were covered by commercial or Medicare Advantage insurance plans; therefore, the results may not be generalizable to patients with other insurance types, such as Medicaid, the overall Medicare covered population, and those without health insurance coverage.

Compensating strengths of this study include its use of a large administrative claims database of patients from geographically dispersed U.S. health plans to study all hepatitis vaccines. In addition to completion, this study also assessed adherence, in contrast to most studies of vaccine compliance. Lastly, the inclusion of data from 2010–2020 enabled study of a very large number of subjects from across the U.S., including the two dose HepB vaccine which was FDA-approved in November 2017, allowing us to derive more generalizable findings than previous studies. As such, this study contributes to the existing limited evidence base on vaccine adherence and completion rates and elucidates factors associated with poor coverage, adherence, and completion rates in the U.S., including for several population groups that are adversely impacted by healthcare disparities.

## 5. Conclusions

Among a geographically representative sample of US adults, adherence and completion rates are low for all hepatitis vaccines, with significant variation by sociodemographic and clinical characteristics. Risk for low adherence and completion was generally associated with male gender, younger age, Black or Hispanic race/ethnicity, and lower educational and household income status. The present study presents important, novel, and timely information which may be used to help strengthen current vaccination strategies. Additional research is needed to uncover factors contributing to poor adherence or completion in order to further inform health policy and programs. However, these findings also serve as a salutary reminder for healthcare workers and organizations of the importance of patient follow-up to improve vaccine uptake and compliance, especially among vulnerable populations.

## Supporting information

S1 Table Vaccine CPT and NDC codes. (DOCX)

S2 Table Quan-Charlson Comorbidity Index. (DOCX)

S3 Table 24-month completion rates among US adults vaccinated with Hepatitis A or B. (DOCX)

S4 Table Socio-demographic and clinical characteristics of completion cohorts. (DOCX)

S5 Table Sensitivity analyses- with baseline period extended to 12 months. (DOCX)

S6 Table Adherence and completion rates for HepA, HepB3 and HepAB initiators with chronic comorbidities. (DOCX)

S7 Table Logistic regression—impact of socio-demographics and clinical/behavioral related variables on adherence. (DOCX)

S8 Table Logistic regression- impact of socio-demographics and clinical/behavioral related variables on completion. (DOCX)
