# Supplementary material for: Hepatitis vaccination adherence and completion rates and factors associated with low compliance: A claims-based analysis of U.S. adults
Source: PLoS One. 2022 Feb 17;17(2):e0264062. doi: 10.1371/journal.pone.0264062 (PMC8853527; doi:10.1371/journal.pone.0264062)
Supplement: S1 Table — (DOCX) [file pone.0264062.s001.docx]

**S1 Table. Vaccine CPT and NDC Codes.**

| **Product name (FDA approval year)** | **Type** | **Doses** | **CPT/NDC Codes** |
| --- | --- | --- | --- |
| Vaqta (1996) | HepA | 2 | 90632 |
| Havrix (1995) | HepA | 2 | 90632 |
| Twinrix (2001) | HepAB | 3 | 90636 |
| Recombivax HB (1986) | HepB | 3 | 90746, 90744 |
| Heplisav-B (2017) | HepB | 2 | 90739 |
| Engerix B (1989) | HepB | 3 | 90746, 90744 |

CPT=Current Procedural Terminology; FDA=Food and Drug Administration; Hep=hepatitis; NDC=National Drug Code.

References: <https://www.cms.gov/Medicare/Medicare-Fee-for-Service-Payment/PhysicianFeeSched/PFS-Relative-Value-Files> Accessed June 22, 2021.
