# Supplementary material for: Hepatitis vaccination adherence and completion rates and factors associated with low compliance: A claims-based analysis of U.S. adults
Source: PLoS One. 2022 Feb 17;17(2):e0264062. doi: 10.1371/journal.pone.0264062 (PMC8853527; doi:10.1371/journal.pone.0264062)
Supplement: S2 Table — (DOCX) [file pone.0264062.s002.docx]

**S2 Table. Quan-Charlson Comorbidity Index.**

| Comorbidities | ICD-9-CM Codes* | ICD-10-CM Codes* | Weight** |
| --- | --- | --- | --- |
| Myocardial infarction | 410.%, 412.% | I21.%, I22.%, I25.2% | 0 |
| Congestive heart failure | 398.91, 402.01, 402.11, 402.91, 404.01, 404.03, 404.11, 404.13, 404.91, 404.93, 425.4%-425.9%, 428.% | I09.9%, I11.0%, I13.0%, I13.2%, I25.5%, I42.0%, I42.5%–I42.9%, I43.%, I50.%, P29.0% | 2 |
| Peripheral vascular disease | 093.0%, 437.3%, 440.%, 441.%, 443.1%-443.9%, 447.1% 557.1%, 557.9%, V43.4% | I70.%, I71.%, I73.1%, I73.8%, I73.9%, I77.1%, I79.0%, I79.2%, K55.1%, K55.8%, K55.9%, Z95.8%, Z95.9% | 0 |
| Cerebrovascular disease | 362.34, 430.% – 438.% | G45.%, G46.%, H34.0%, I60.%–I69.% | 0 |
| Dementia | 290.%, 294.1%, 331.2% | F00.%–F03.%, F05.1, G30.%, G31.1% | 2 |
| Chronic Pulmonary Disease | 416.8%, 416.9%, 490.%-505.%, 506.4%, 508.1%, 508.8% | I27.8%, I27.9%, J40.%–J47.%, J60.%–J67.%, J68.4%, J70.1%, J70.3% | 1 |
| Connective tissue/rheumatic disease | 446.5%, 710.0% – 710.4%, 714.0% – 714.2%, 714.8%, 725.% | M05.%, M06.%, M31.5%, M32.%–M34.%, M35.1%, M35.3%, M36.0% | 1 |
| Peptic ulcer disease | 531.% – 534.% | K25.%–K28.% | 0 |
| Mild liver disease: | 070.22, 070.23, 070.32, 070.33, 070.44, 070.54, 070.6%, 070.9%, 570.%, 571.%, 573.3%, 573.4%, 573.8%, 573.9%, V42.7% | B18.%, K70.0%–K70.3%, K70.9%, K71.3%–K71.5%, K71.7%, K73.%, K74.%, K76.0%, K76.2%–K76.4%, K76.8%, K76.9%, Z94.4% | 2 |
| Diabetes without chronic complications/mild to moderate | 250.0% – 250.3%; 250.8%, 250.9% | E10.0%, E10.1%, E10.6%, E10.8%, E10.9%, E11.0%, E11.1%, E11.6%, E11.8%, E11.9%, E12.0%, E12.1%, E12.6%, E12.8%, E12.9%, E13.0%, E13.1%, E13.6%, E13.8%, E13.9%, E14.0%, E14.1%, E14.6%, E14.8%, E14.9% | 0 |
| Paraplegia and hemiplegia | 334.1%, 342.%, 343.%, 344.0%-344.6%, 344.9% | G04.1%, G11.4%, G80.1%, G80.2%, G81.%, G82.%, G83.0%–G83.4%, G83.9% | 2 |
| Renal disease | 403.01, 403.11, 403.91, 404.02, 404.03, 404.12, 404.13, 404.92, 404.93, 582.0% – 583.7%, 585.%, 586.%, 588.0%, V42.0%, V45.1%, V56.% | I12.0%, I13.1%, N03.2%–N03.7%, N05.2%–N05.7%, N18.%, N19.%, N25.0%, Z49.0%–Z49.2%, Z94.0%, Z99.2% | 1 |
| Diabetes with chronic complications | 250.4% – 250.7% | E10.2%–E10.5%, E10.7%, E11.2%–E11.5%, E11.7%, E12.2%–E12.5%, E12.7%, E13.2%–E13.5%, E13.7%, E14.2%–E14.5%, E14.7% | 1 |
| Any malignancy, including lymphoma and leukemia, except malignant neoplasm of skin | 140.% – 172.%, 174.% – 195.8%, 200.% – 208.%, 238.6% | C00.%–C26.%, C30.%–C34.%, C37.%–C41.%, C43.%, C45.%–C58.%, C60.%–C76.%, C81.%–C85.%, C88.%, C90.%–C97.% | 2 |
| Moderate or severe liver disease | 456.0%-456.2%, 572.2%-572.8% | I85.0%, I85.9%, I86.4%, I98.2%, K70.4%, K71.1%, K72.1%, K72.9%, K76.5%, K76.6%, K76.7% | 4 |
| Metastatic solid tumor | 196.% – 199.% | C77.%–C80.% | 6 |
| AIDS/HIV | 042.%, 043.%, 044.% | B20.%–B22.%, B24.% | 4 |

To quantify comorbidity, the QCI score is computed by adding the weights that are assigned to the specific diagnoses. Each diagnosis is only counted once. The minimum possible score is 0 and the maximum possible score is 24.

**References:** * Quan et al. Coding algorithms for defining comorbidities in ICD-9-CM and ICD-10 administrative data. Med Care. 2005 Nov;43(11):1130-9.

** Quan et al. Updating and Validating the Charlson Comorbidity Index and Score for Risk Adjustment in Hospital Discharge Abstracts Using Data From six Countries. Am J Epidemiol. 2011. Mar 15;173(6):676-82.
