# Supplementary material for: Hepatitis vaccination adherence and completion rates and factors associated with low compliance: A claims-based analysis of U.S. adults
Source: PLoS One. 2022 Feb 17;17(2):e0264062. doi: 10.1371/journal.pone.0264062 (PMC8853527; doi:10.1371/journal.pone.0264062)
Supplement: S3 Table — (DOCX) [file pone.0264062.s003.docx]

**S3 Table. 24-month completion rates among US adults vaccinated for Hepatitis A or B.**

|  | Hep A (2 doses) | Hep B (2 doses) | Hep B (3 doses) | Hep AB (3 doses) |
| --- | --- | --- | --- | --- |
|  | N=75,561 | N=134 | N=99,560 | N=34,925 |
| Overall | 28.3% | 44.8% | 37.3% | 33.8% |
| Gender | ^***^ |  |  | ^***^ |
| Male | 27.7% | 48.4% | 37.1% | 29.5% |
| Female | 28.9% | 61.1% | 37.5% | 37.4% |
| Age group | ^***^ |  | ^***^ | ^***^ |
| 18-39 | 23.7% | 62.1% | 29.8% | 31.0% |
| 40-64 | 33.2% | 51.6% | 41.0% | 38.1% |
| 65-74 | 28.0% | 56.8% | 41.9% | 18.9% |
| ≥75 | 23.7% | 50.0% | 41.3% | 15.3% |
| Race/ethnicity | ^***^ |  | ^***^ | ^***^ |
| Asian | 29.5% | 40.0% | 43.8% | 28.8% |
| Black | 24.7% | 57.1% | 33.5% | 34.7% |
| Hispanic | 25.9% | 76.5% | 35.1% | 33.3% |
| White | 28.8% | 51.9% | 37.4% | 34.4% |
| Unknown/other | 27.7% | 57.1% | 36.5% | 29.6% |
| Region | ^***^ |  | ^***^ | ^***^ |
| Northeast | 27.4% | 58.3% | 40.5% | 28.1% |
| Midwest | 29.8% | 45.8% | 37.8% | 30.7% |
| South | 27.3% | 63.0% | 36.7% | 40.4% |
| West | 28.2% | 51.9% | 36.4% | 26.3% |
| Household income | ^***^ | ^*^ | ^***^ | ^***^ |
| <$40k | 28.3% | 29.4% | 36.1% | 33.1% |
| $40k-60k | 27.3% | 75.0% | 37.3% | 35.9% |
| $60k-100k | 29.1% | 32.0% | 39.2% | 36.9% |
| $100k + | 28.6% | 47.8% | 37.7% | 33.5% |
| Unknown | 26.8% | 47.1% | 35.2% | 29.3% |
| Education level (census block level) |  |  | ^*^ | ^**^ |
| ≤ High School | 29.5% | 46.2% | 36.6% | 40.2% |
| > High School | 28.2% | 44.1% | 37.6% | 32.5% |
| Unknown | 27.4% | 46.7% | 36.3% | 27.5% |
| Insurance type | ^***^ |  | ^***^ | ^***^ |
| Commercial | 28.7% | 42.3% | 36.3% | 36.6% |
| Medicare | 25.8% | 48.2% | 41.1% | 13.5% |
| CCI condition | ^***^ |  | ^***^ | ^***^ |
| 0 | 27.6% | 44.0% | 35.6% | 34.3% |
| 1-2 | 30.8% | 40.0% | 42.5% | 33.8% |
| ≥3 | 31.5% | 60.0% | 41.8% | 28.7% |
| ER visit – baseline | 28.8% | 37.5% | 36.0% | 34.7% |
| In patient -baseline | 28.5% | 33.3% | 36.0% | 32.6% |
| Provider type for first dose | ^***^ |  | ^***^ | ^***^ |
| Family practice | 29.6% | 44.2% | 37.1% | 34.2% |
| Internal medicine | 29.9% | 46.9% | 41.8% | 31.4% |
| Nursing service providers | 24.5% | 25.0% | 31.9% | 30.8% |
| Pharmacy service providers | 14.3% | NA | 37.7% | 36.6% |
| Infectious diseases | 30.7% | NA | 35.6% | 34.5% |
| Others | 24.9% | 52.9% | 33.2% | 35.2% |

Note: results of HepB2 dose may not be representative due to small sample size.

*** p<0.001; ** p<0.01, *<0.05 from Chi-square.
