# Supplementary material for: Hepatitis vaccination adherence and completion rates and factors associated with low compliance: A claims-based analysis of U.S. adults
Source: PLoS One. 2022 Feb 17;17(2):e0264062. doi: 10.1371/journal.pone.0264062 (PMC8853527; doi:10.1371/journal.pone.0264062)
Supplement: S4 Table — (DOCX) [file pone.0264062.s004.docx]

**S4 Table.** **Socio-demographic and Clinical Characteristics of Completion Cohorts (those with 24 months of follow-up).**

|  | | | | | | | | |
| --- | --- | --- | --- | --- | --- | --- | --- | --- |
|  | Hep A (2 doses) | | Hep B (2 doses) | | Hep B (3 doses) | | Hep AB (3 doses) | |
|  | N |  | N |  | N |  | N |  |
| Overall | 75561 |  | 134 |  | 99560 |  | 34925 |  |
| Gender |  |  |  |  |  |  |  |  |
| Male | 35193 | 46.6% | 62 | 46.3% | 46196 | 46.4% | 15736 | 45.1% |
| Female | 40368 | 53.4% | 72 | 53.7% | 53364 | 53.6% | 19189 | 54.9% |
| Age group |  |  |  |  |  |  |  |  |
| 18-39 | 31423 | 41.6% | 29 | 21.6% | 33564 | 33.7% | 9672 | 27.7% |
| 40-64 | 33345 | 44.1% | 62 | 46.3% | 47583 | 47.8% | 21241 | 60.8% |
| 65-74 | 7932 | 10.5% | 37 | 27.6% | 14185 | 14.2% | 3280 | 9.4% |
| ≥75 | 2861 | 3.8% | 6 | 4.5% | 4228 | 4.2% | 732 | 2.1% |
| Race/ethnicity |  |  |  |  |  |  |  |  |
| Asian | 6106 | 8.1% | 10 | 7.5% | 9914 | 10.0% | 1945 | 5.6% |
| Black | 4791 | 6.3% | 7 | 5.2% | 9407 | 9.4% | 3662 | 10.5% |
| Hispanic | 5545 | 7.3% | 17 | 12.7% | 12443 | 12.5% | 3127 | 9.0% |
| White | 55300 | 73.2% | 79 | 59.0% | 62253 | 62.5% | 24679 | 70.7% |
| Unknown/other | 3819 | 5.1% | 21 | 15.7% | 5543 | 5.6% | 1512 | 4.3% |
| Region |  |  |  |  |  |  |  |  |
| Northeast | 9310 | 12.3% | 12 | 9.0% | 13218 | 13.3% | 2847 | 8.2% |
| Midwest | 24460 | 32.4% | 24 | 17.9% | 24622 | 24.7% | 8017 | 23.0% |
| South | 20525 | 27.2% | 46 | 34.3% | 34619 | 34.8% | 15912 | 45.6% |
| West | 21141 | 28.0% | 52 | 38.8% | 26844 | 27.0% | 8120 | 23.2% |
| Unknown | 125 | 0.2% | 0 | 0.0% | 257 | 0.3% | 29 | 0.1% |
| Household income |  |  |  |  |  |  |  |  |
| <$40k | 6511 | 8.6% | 17 | 12.7% | 14740 | 14.8% | 4530 | 13.0% |
| $40k-60k | 5760 | 7.6% | 12 | 9.0% | 10510 | 10.6% | 3571 | 10.2% |
| $60k-100k | 14901 | 19.7% | 25 | 18.7% | 21021 | 21.1% | 7601 | 21.8% |
| $100k + | 37738 | 49.9% | 46 | 34.3% | 36947 | 37.1% | 14329 | 41.0% |
| Unknown | 10651 | 14.1% | 34 | 25.4% | 16342 | 16.4% | 4894 | 14.0% |
| Education level (census block level) | |  |  |  |  |  |  |  |
| ≤ High School | 7998 | 10.6% | 26 | 19.4% | 18580 | 18.7% | 6570 | 18.8% |
| > High School | 65657 | 86.9% | 93 | 69.4% | 77639 | 78.0% | 27502 | 78.7% |
| Unknown | 1906 | 2.5% | 15 | 11.2% | 3341 | 3.4% | 853 | 2.4% |
| Insurance type |  |  |  |  |  |  |  |  |
| Commercial | 65660 | 86.9% | 78 | 58.2% | 77967 | 78.3% | 30775 | 88.1% |
| Medicare | 9901 | 13.1% | 56 | 41.8% | 21593 | 21.7% | 4150 | 11.9% |
| CCI condition |  |  |  |  |  |  |  |  |
| 0 | 58319 | 77.2% | 109 | 81.3% | 73469 | 73.8% | 27523 | 78.8% |
| 1-2 | 12861 | 17.0% | 15 | 11.2% | 17717 | 17.8% | 5050 | 14.5% |
| ≥3 | 4381 | 5.8% | 10 | 7.5% | 8374 | 8.4% | 2352 | 6.7% |
| ER visit - baseline | 18696 | 24.7% | 8 | 6.0% | 13333 | 13.4% | 4700 | 13.5% |
| In patient -baseline | 6636 | 8.8% | 3 | 2.2% | 6603 | 6.6% | 1898 | 5.4% |
| Provider type for first dose | | |  |  |  |  |  |  |
| Family practice | 34660 | 45.9% | 77 | 57.5% | 47927 | 48.1% | 15718 | 45.0% |
| Internal medicine | 16851 | 22.3% | 32 | 23.9% | 24568 | 24.7% | 7257 | 20.8% |
| Nursing | 2268 | 3.0% | 8 | 6.0% | 2282 | 2.3% | 869 | 2.5% |
| Pharmacy | 84 | 0.1% | 0 | 0.0% | 2916 | 2.9% | 435 | 1.2% |
| Infectious disease | 2901 | 3.8% | 0 | 0.0% | 1727 | 1.7% | 1264 | 3.6% |
| Others | 18793 | 24.9% | 17 | 12.7% | 20139 | 20.2% | 9382 | 26.9% |
| Unknown | 4 | 0.0% | 0 | 0.0% | 1 | 0.0% | 0 | 0.0% |
