# Supplementary material for: Hepatitis vaccination adherence and completion rates and factors associated with low compliance: A claims-based analysis of U.S. adults
Source: PLoS One. 2022 Feb 17;17(2):e0264062. doi: 10.1371/journal.pone.0264062 (PMC8853527; doi:10.1371/journal.pone.0264062)
Supplement: S5 Table — (DOCX) [file pone.0264062.s005.docx]

**S5 Table. Sensitivity analyses using baseline period extended to 12 months.**

|  | Sensitivity analyses | | |
| --- | --- | --- | --- |
|  | Baseline (months) | N | Adherence rate |
| Hep B (2 doses) | 12 | 5,063 | 32.75% |
| Hep B (3 doses) | 12 | 151,959 | 14.81% |
| Hep AB (3 doses) | 12 | 51,229 | 15.93% |
