# Supplementary material for: Hepatitis vaccination adherence and completion rates and factors associated with low compliance: A claims-based analysis of U.S. adults
Source: PLoS One. 2022 Feb 17;17(2):e0264062. doi: 10.1371/journal.pone.0264062 (PMC8853527; doi:10.1371/journal.pone.0264062)
Supplement: S6 Table — (DOCX) [file pone.0264062.s006.docx]

**S6 Table. Adherence and completion rates for HepA, HepB3 and HepAB initiators with chronic comorbidities.**

|  | Hep A | | | | | | Hep B3 | | | | | | Hep AB | | | | | |
| --- | --- | --- | --- | --- | --- | --- | --- | --- | --- | --- | --- | --- | --- | --- | --- | --- | --- | --- |
|  | adherence | | | completion (24 months) | | | adherence | | | completion (24 months) | | | adherence | | | completion (24 months) | | |
|  | N | % | p value | N | % | p value | N | % | p value | N | % | p value | N | % | p value | N | % | p value |
| Overall | 93986 | 27.0 | - | 75561 | 28.4 | - | 191761 | 14.3 | - | 99560 | 37.3 | - | 64286 | 15.3 | - | 34925 | 33.8 | - |
| CCI condition |  |  |  |  |  |  |  |  |  |  |  |  |  |  |  |  |  |  |
| Diabetes with chronic complications | 2391 | 27.0 | 0.98 | 1812 | 27.8 | 0.58 | 10394 | 13.8 | 0.15 | 5207 | 44.5 | <.0001 | 1714 | 9.9 | <.0001 | 799 | 25.0 | <.0001 |
| Diabetes without chronic complications/ mild-moderate | 7534 | 28.9 | <.0001 | 5868 | 29.8 | 0.01 | 32065 | 14.8 | 0.01 | 16859 | 45.5 | <.0001 | 6673 | 12.9 | <.0001 | 3421 | 31.6 | 0.004 |
| Mild liver disease | 5834 | 34.4 | .0001 | 4657 | 35.7 | <.0001 | 18545 | 18.3 | <.0001 | 9653 | 43.4 | <.0001 | 6361 | 16.1 | 0.07 | 3277 | 32.3 | 0.052 |
| Moderate or severe liver disease | 752 | 30.2 | 0.04 | 569 | 32.0 | 0.062 | 3269 | 16.6 | .0001 | 1583 | 40.7 | 0.006 | 1068 | 13.9 | 0.13 | 508 | 26.2 | 0.0002 |
| Renal disease | 2607 | 27.8 | 0.3 | 2068 | 29.1 | 0.489 | 8466 | 15.4 | 0.003 | 4437 | 45.1 | <.0001 | 1481 | 10.4 | <.0001 | 735 | 25.9 | <.0001 |
