# Supplementary material for: Hepatitis vaccination adherence and completion rates and factors associated with low compliance: A claims-based analysis of U.S. adults
Source: PLoS One. 2022 Feb 17;17(2):e0264062. doi: 10.1371/journal.pone.0264062 (PMC8853527; doi:10.1371/journal.pone.0264062)
Supplement: S7 Table — (DOCX) [file pone.0264062.s007.docx]

**S7 Table. Logistic regression- Impact of sociodemographic and clinical/behavioral variables on adherence to vaccine schedules for Hepatitis A or B.**

|  | HepA (N=93,986) | | | HepB2 (N=6,795) | | | HepB3 (N=191,761) | | | HepAB (N=64,286) | | | |  |
| --- | --- | --- | --- | --- | --- | --- | --- | --- | --- | --- | --- | --- | --- | --- |
|  | OR | 95%CI | | OR | 95%CI | | OR | 95%CI | | OR | 95%CI | | |  |
| Age group |  |  |  |  |  |  |  |  |  |  |  |  | | |
| 40-64 vs 18-39 | 1.64 | 1.58 | 1.69 | 0.98 | 0.86 | 1.12 | 1.42 | 1.38 | 1.47 | 1.45 | 1.38 | | 1.52 | |
| 65-74 vs 18-39 | 1.55 | 1.44 | 1.67 | 0.89 | 0.72 | 1.11 | 1.62 | 1.52 | 1.72 | 1.48 | 1.32 | | 1.67 | |
| >75 vs 18-39 | 1.30 | 1.17 | 1.45 | 1.06 | 0.81 | 1.38 | 1.66 | 1.53 | 1.81 | 1.53 | 1.17 | | 1.98 | |
| Commercial vs Medicare | 1.48 | 1.37 | 1.60 | 0.95 | 0.79 | 1.15 | 1.09 | 1.04 | 1.15 | 4.53 | 3.92 | | 5.22 | |
| CCI score |  |  |  |  |  |  |  |  |  |  |  | |  | |
| 1-2 vs 0 | 1.16 | 1.12 | 1.21 | 0.99 | 0.84 | 1.15 | 1.07 | 1.04 | 1.11 | 1.08 | 1.01 | | 1.15 | |
| ≥3 vs 0 | 1.24 | 1.16 | 1.32 | 1.05 | 0.86 | 1.29 | 1.06 | 1.01 | 1.12 | 0.92 | 0.83 | | 1.01 | |
| > High School Diploma vs ≤ High School Diploma | 0.82 | 0.78 | 0.86 | 1.18 | 1.01 | 1.37 | 1.07 | 1.03 | 1.11 | 0.95 | 0.90 | | 1.01 | |
| Female vs Male | 1.08 | 1.05 | 1.11 | 1.11 | 1.00 | 1.24 | 1.12 | 1.09 | 1.15 | 1.26 | 1.21 | | 1.32 | |
| Household income |  |  |  |  |  |  |  |  |  |  |  | |  | |
| $40-60k vs <$40k | 0.98 | 0.92 | 1.06 | 1.21 | 0.97 | 1.51 | 1.13 | 1.07 | 1.19 | 1.13 | 1.02 | | 1.24 | |
| $60-100k vs <$40k | 0.98 | 0.91 | 1.05 | 1.43 | 1.12 | 1.81 | 1.19 | 1.12 | 1.26 | 1.25 | 1.13 | | 1.38 | |
| ≥100k vs <$40k | 0.96 | 0.91 | 1.01 | 1.15 | 0.96 | 1.38 | 1.12 | 1.08 | 1.17 | 1.10 | 1.02 | | 1.18 | |
| Provider type for 1st dose |  |  |  |  |  |  |  |  |  |  |  | |  | |
| Internal medicine vs Family practice | 1.02 | 0.98 | 1.06 | 0.82 | 0.63 | 1.08 | 1.36 | 1.32 | 1.40 | 1.35 | 1.27 | | 1.43 | |
| Nursing facility vs Family practice | 0.78 | 0.71 | 0.85 | 0.99 | 0.75 | 1.30 | 0.95 | 0.87 | 1.04 | 1.15 | 1.00 | | 1.32 | |
| Pharmacist vs Family practice | 1.20 | 0.99 | 1.46 | 0.95 | 0.66 | 1.36 | 1.18 | 1.11 | 1.26 | 1.24 | 1.11 | | 1.38 | |
| Infectious disease vs Family practice | 1.00 | 0.92 | 1.08 | 1.44 | 0.98 | 2.10 | 1.02 | 0.92 | 1.14 | 1.71 | 1.52 | | 1.93 | |
| Other vs Family practice | 0.85 | 0.81 | 0.88 | 1.03 | 0.78 | 1.36 | 0.90 | 0.87 | 0.94 | 1.59 | 1.51 | | 1.68 | |
| Race/ethnicity |  |  |  |  |  |  |  |  |  |  |  | |  | |
| Asian vs White | 1.02 | 0.97 | 1.08 | 1.04 | 0.85 | 1.29 | 1.30 | 1.25 | 1.36 | 0.94 | 0.85 | | 1.04 | |
| Black vs White | 0.79 | 0.74 | 0.84 | 1.13 | 0.94 | 1.36 | 0.77 | 0.73 | 0.81 | 0.72 | 0.67 | | 0.78 | |
| Hispanic vs White | 0.86 | 0.81 | 0.91 | 1.05 | 0.92 | 1.20 | 0.83 | 0.79 | 0.86 | 0.74 | 0.68 | 0.80 | | |
