# Supplementary material for: Hepatitis vaccination adherence and completion rates and factors associated with low compliance: A claims-based analysis of U.S. adults
Source: PLoS One. 2022 Feb 17;17(2):e0264062. doi: 10.1371/journal.pone.0264062 (PMC8853527; doi:10.1371/journal.pone.0264062)
Supplement: S8 Table — (DOCX) [file pone.0264062.s008.docx]

**S8 Table. Logistic regression- Impact of sociodemographic and clinical/behavioral variables on completion (24 months) of vaccination for Hepatitis A and B.**

|  | HepA (N=75,561)N=75,561 | | | | HepB2 (N=134)N=134 | | | | | HepB3 (N=99,560)N=99,560 | | | HepAB (N=34,925)N=34,925 | | | | |
| --- | --- | --- | --- | --- | --- | --- | --- | --- | --- | --- | --- | --- | --- | --- | --- | --- | --- |
|  | OR | 95%CI | | OR | | 95%CI | | OR | | | 95%CI | | | OR | 95%CI | |  |
| Age group |  |  |  |  | |  |  | |  | |  |  | |  |  |  |  |
| 40-64 vs 18-39 | 1.55 | 1.50 | 1.61 | 0.55 | | 0.23 | 1.34 | | 1.60 | | 1.55 | 1.65 | | 1.38 | 1.31 | 1.46 |  |
| 65-74 vs 18-39 | 1.47 | 1.36 | 1.60 | 1.93 | | 0.66 | 5.68 | | 1.66 | | 1.57 | 1.77 | | 1.28 | 1.12 | 1.45 |  |
| >75 vs 18-39 | 1.21 | 1.08 | 1.36 | 2.47 | | 0.20 | 30.26 | | 1.59 | | 1.46 | 1.72 | | 1.33 | 1.05 | 1.69 |  |
| Commercial vs Medicare | 1.41 | 1.29 | 1.53 | 0.98 | | 0.21 | 3.23 | | 1.05 | | 0.99 | 1.11 | | 3.71 | 3.26 | 4.22 |  |
| CCI score |  |  |  |  | |  |  | |  | |  |  | |  |  |  |  |
| 1-2 vs 0 | 1.15 | 1.10 | 1.20 | 0.83 | | 0.21 | 3.23 | | 1.24 | | 1.20 | 1.29 | | 1.11 | 1.04 | 1.19 |  |
| ≥3 vs 0 | 1.24 | 1.15 | 1.33 | 1.80 | | 0.33 | 9.65 | | 1.25 | | 1.19 | 1.31 | | 1.06 | 0.96 | 1.18 |  |
| > High School Diploma vs ≤ High School Diploma | 0.90 | 0.86 | 0.96 | 0.98 | | 0.21 | 3.23 | | 1.03 | | 0.99 | 1.06 | | 0.81 | 0.76 | 0.86 |  |
| Female vs Male | 1.07 | 1.04 | 1.10 | 0.55 | | 0.23 | 1.34 | | 1.07 | | 1.04 | 1.10 | | 1.33 | 1.27 | 1.40 |  |
| Household income |  |  |  |  | |  |  | |  | |  |  | |  |  |  |  |
| $40-60k vs <$40k | 0.94 | 0.87 | 1.02 | 13.14 | | 1.72 | 100.18 | | 1.07 | | 1.01 | 1.12 | | 1.10 | 0.99 | 1.21 |  |
| $60-100k vs <$40k | 0.98 | 0.90 | 1.06 | 0.73 | | 0.09 | 5.92 | | 1.16 | | 1.09 | 1.23 | | 1.13 | 1.02 | 1.25 |  |
| ≥100k vs <$40k | 0.96 | 0.91 | 1.02 | 2.85 | | 0.69 | 11.75 | | 1.08 | | 1.03 | 1.12 | | 0.99 | 0.92 | 1.07 |  |
| Provider type for first dose |  |  |  |  | |  |  | |  | |  |  | |  |  |  |  |
| Internal medicine vs Family practice | 1.00 | 0.96 | 1.05 | 1.01 | | 0.39 | 2.67 | | 1.15 | | 1.11 | 1.19 | | 0.98 | 0.92 | 1.05 |  |
| Nursing facility vs Family practice | 0.76 | 0.68 | 0.83 | 0.41 | | 0.06 | 2.85 | | 0.85 | | 0.77 | 0.93 | | 0.84 | 0.72 | 0.98 |  |
| Pharmacist vs Family practice | 0.37 | 0.20 | 0.68 | NA | | NA | NA | | 1.07 | | 0.99 | 1.16 | | 1.03 | 0.84 | 1.26 |  |
| Infectious disease vs Family practice | 1.00 | 0.92 | 1.09 | NA | | NA | NA | | 0.86 | | 0.78 | 0.96 | | 1.09 | 0.96 | 1.23 |  |
| Other vs Family practice | 0.84 | 0.81 | 0.88 | 1.88 | | 0.51 | 6.91 | | 0.84 | | 0.81 | 0.87 | | 1.02 | 0.96 | 1.08 |  |
| Race/ethnicity |  |  |  |  | |  |  | |  | |  |  | |  |  |  |  |
| Asian vs White | 1.05 | 0.99 | 1.11 | 1.63 | | 0.37 | 7.11 | | 1.37 | | 1.31 | 1.43 | | 0.85 | 0.77 | 0.95 |  |
| Black vs White | 1.47 | 1.36 | 1.60 | 0.81 | | 0.11 | 5.82 | | 0.84 | | 0.80 | 0.88 | | 0.81 | 0.74 | 0.87 |  |
| Hispanic vs White | 1.21 | 1.08 | 1.36 | 0.14 | | 0.03 | 0.66 | | 0.94 | | 0.90 | 0.98 | | 0.90 | 0.83 | 0.98 |  |
